# Too White Flour

**Published:** 1857-03

**Authors:** 


### TOO WHITE FLOUR.

Messrs. Mouriez & Chevrene, chemists, who have superintended the provision of bread for the hospitals, and subjected all kinds to experiments, submitted a report to the French Academy, in which they condemn the practice of making bread *too white.* It is then, they remark, a condiment, not an aliment. The exclusion of bran is a loss of nourishment to the consumer; the palate and fancy are gratified at the expense of the whole animal economy.

